# Practical introduction of novel oral anticoagulants through an anticoagulation nurse. The Leeuwarden model

**DOI:** 10.1007/s12471-014-0529-9

**Published:** 2014-03-04

**Authors:** R. J. Folkeringa, L. M. Geven, T. Veldhuis, M. Hoogendoorn, S. H. Hofma, E. Van Roon

**Affiliations:** 1 Department of Cardiology, Medical Centre Leeuwarden, Henry Dunantweg 2, 8934 AD Leeuwarden, the Netherlands; 2 Thrombosis service Friesland Noord, Borniastraat 34, 8934 AD Leeuwarden, the Netherlands; 3 Department of Internal Medicine, Medical Centre Leeuwarden, Henry Dunantweg 2, 8934 AD Leeuwarden, the Netherlands; 4 Department of Pharmacy, Medical Centre Leeuwarden, Henry Dunantweg 2, 8934 AD Leeuwarden, the Netherlands

**Keywords:** Atrial fibrillation, Novel oral anticoagulants, Anticoagulation

## Abstract

**Introduction:**

This paper describes the implementation of novel oral anticoagulants (NOACs) through an anticoagulation nurse. Logistics and tasks of this new function are described and preliminary data are presented.

**Methods:**

Indications for NOACs are explained by the treating cardiologists. Thereafter, the patient is referred to the anticoagulation nurse before starting a NOAC. After providing a patient with information and checking the creatinine clearance, co-medication and medical history, a prescription for NOAC is made.

**Results:**

In 3 months, 51 patients were referred for NOAC therapy. Mean age was 68 years, CHA2DS2-VASc score was 2.9. Renal function was impaired in 28 %. Only 63 % of the patients had an uneventful start-up. NOAC therapy was withheld or prematurely stopped in 22 %. 30 % of patients needed a reduced NOAC dose. In 37 %, the anticoagulation nurse had extended patient contact, mainly because of (presumed) side effects.

**Conclusion:**

Given the number of interactions that were made using a separate patient contact through the anticoagulation nurse, this seems to be an important improvement in the quality of care and deserves further expansion.

## Introduction

European guidelines state a preference for novel oral anticoagulants (NOACs) above vitamin K antagonists (VKA) for the indication non-valvular atrial fibrillation (AF) [1, 2]. In the Netherlands, the Ministry of Health ordered measures to be taken for the safe prescription of these drugs by professionals, which led to the Guideline for the introduction of new oral anticoagulants, known as the Leidraad [3]. This paper describes the implementation of the ‘Leidraad’ in The Medical Centre Leeuwarden, thereby creating a new function for the anticoagulation nurse. Preliminary data are presented of our first experience with NOACs using this format.

## Methods

### The anticoagulation nurse

In order to secure ‘chain care’, a new function of the anticoagulation nurse was launched in close collaboration with the Thrombosis Service, Department of Internal Medicine and the Hospital Pharmacy.

During an outpatient visit, indications for using anticoagulants are explained by the treating cardiologists but a profound discussion over the benefits and disadvantages of using a NOAC is usually lacking. Therefore, in our hospital, the patient is referred to the anticoagulation nurse before starting a NOAC.

After providing a patient with information and checking the creatinine clearance, co-medication and medical history, a prescription for NOAC and authorisation form for the health insurance company is produced. A supervising cardiologist controls the checklist and signs the prescription and authorisation form. A standard letter mentions the indication, drug and dose, comorbidity, creatinine clearance, other drugs and the CHA_2_DS_2_-VASc and HASBLED scores.

Data regarding compliance, side effects, and complications are marked in a central database. A major advantage of the anticoagulation nurse is that all patients potentially receiving a NOAC are identified centrally. In this way, the NOAC prescriber, in this case the cardiologist, is able to take adequate control over this new therapy.

## Results

The anticoagulation nurse started in February 2013. After 3 months, 51 patients have been referred for NOAC therapy; mean age was 68 years, 65 % males (Table 1).

**Table 1:** Baseline characteristics

Characteristics	*N* = 51 (%)
Age, year	68
Male, *n* (%)	33 (65)
eGRF ≥60 ml/min	37 (73)
- 50–60 ml/min	6 (12)
- <50 ml/min	8 (16)
CHA_2_DS_2_-VASc score	2.9
HASBLED score	1.9
VKA naive	20 (39)
Switch from VKA	31 (61)
Main reason for switching to NOAC	
- User friendly	4/31 (13)
- Dislike frequent blood sample taking	6 (19)
- Side effects	2 (6)
- Cardiologist’s advice	4 (13)
- Labile INR (according to patient)	4 (13)
- After use in trial	1 (3)
- Unknown or no specific reason	10 (32)
Dabigatran dose	
- 2 dd 150 mg	8 (18)*
- 2 dd 110 mg	9 (20)*
Rivaroxaban dose	
- 1 dd 20 mg	22 (51)*
- 1 dd 15 mg	4 (9)*
Reason reduced NOAC dose	
- Reduced creatinine clearance	8 (62)
- Age, years	2 (15)
- Drug interaction	2 (15)
- Around PVI	1 (8)

*Referred to 43 patients, *n* = 43, after exclusion of 8 patients who were not prescribed with an NOAC

*NOAC* novel oral anticoagulants; *PVI* pulmonary vein isolation; *VKA* vitamin K antagonists

After consultation with the anticoagulation nurse, in 8/51 patients (16 %) no NOAC was prescribed: two patients were considered to lack an indication for oral anticoagulation, because of lack of registration of AF and left atrial appendix amputation during prior cardiac surgery, respectively. Three patients refused NOAC because of negative publicity. In another three patients, NOAC therapy was contraindicated because of concomitant severe gastrointestinal (including stomach) symptoms or poor renal function. Of the 43 patients who started on a NOAC, 13 (30 %) were treated with a reduced dose, mainly because of abnormal renal function, defined as eGFR ≤50 ml/min.

**Fig. 1:**
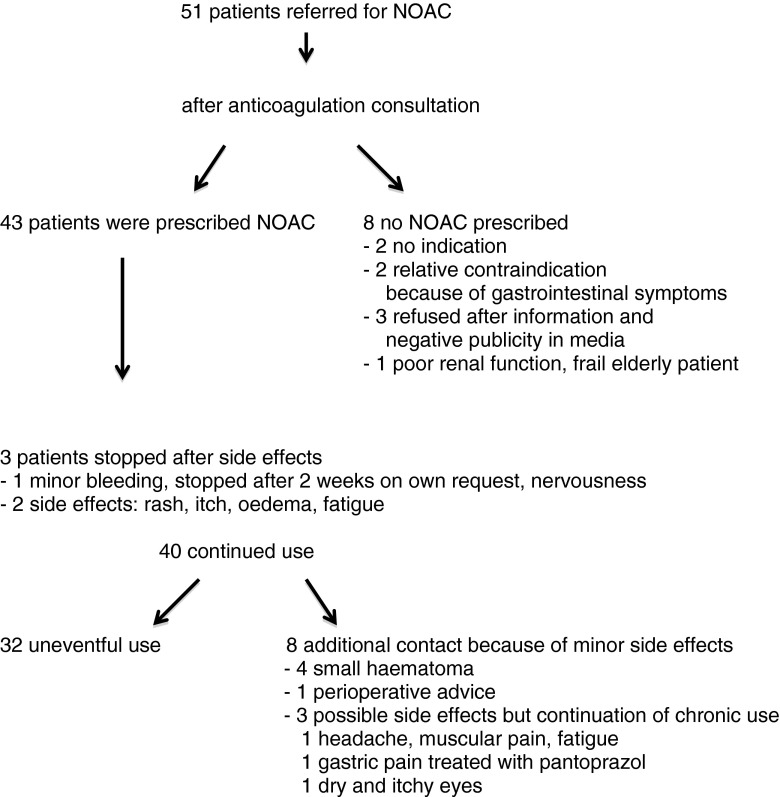
Flowchart of patients

The anticoagulation nurse had additional contacts with 11 of 43 patients (26 %). In three patients, NOAC therapy was stopped within 2 weeks because of side effects. One patient developed rash, itch and oedema. One patient suffered from fatigue and one patient had a minor bleed, and refused further NOAC therapy because of lack of an antidote. Four had minor bleeding and one bridging advice was given. In three patients, possible side effects were reported, but drug continuation was advised. Figure 1 shows the flowchart of patients during the study.

In conclusion, after referral, 32/51 (63 %) of the patients had an uneventful start-up of NOAC therapy. NOAC therapy was withheld or prematurely stopped in 22 %. A considerable number of patients needed a reduced NOAC dose. In 37 %, the coagulation nurse had extended patient contact, mainly because of (presumed) side effects.

## Discussion

This pilot study describes the logistics and first experience of a new function of anticoagulation nurse. Although a central role for pharmacists has been proposed by ‘De Leidraad’, in our model cardiologists are comprehensively facilitated in keeping control over NOAC therapy. In this way they can fully comply with their role as prescriber of anticoagulation.

The main finding of our observations is that an anticoagulation nurse is a feasible and valuable intermediate between the cardiologist and the patient. These data suggest that introducing a NOAC is labour intensive and an additional patient contact is pivotal for safety and education. The anticoagulant nurse is easily accessible for patients and helps with additional tasks such as registration of complications. [4].

Recently, the Ministry of Health has strongly encouraged the use of a case manager for oral anticoagulants [4]. The function of the anticoagulation nurse can be extended to a case manager for all patients using oral anticoagulants.

### Safety of NOAC’s: research versus daily practice

The most important requirement before starting prescribing a NOAC is the organisation of ‘chain care’ [3–5]. During a randomised clinical trial (RCT), this is done by the research institute and dictated by a strict protocol. Frequent contacts and patient education may bias towards more safety in RCTs. A run-in phase of a trial usually excludes non-compliant patients as well as patients prone to side effects. The anticoagulation nurse helps to prevent prescription errors which is an important determinant of iatrogenic bleeding [6]. In our small cohort, it was decided to withhold a NOAC in 8 patients after extended discussion. We believe, especially in the introduction phase, that intensive consideration of the indication for a NOAC is necessary to preclude prescription to the ‘frail elderly’ as well as potentially non-compliant patients.

### Major vs minor bleeding

Compared with stroke and major bleeding, patients much more frequently suffer from minor side effects, such as minor bleeding or dyspepsia. Every year, minor bleeding may occur in 1 out of 6–7 patients. Within 2 years, 1 out of 4–5 patients will discontinue NOAC therapy [7–9]. In these patients, a transoesophageal echocardiography-guided strategy may be useful since it may help to avoid anticoagulation in apparently low-risk patients with non-valvular AF [10].

Our data suggest that after prescribing a NOAC, it is expected that caregivers will have contact with the patient much more often than anticipated. The Thrombosis Service represents a robust network and takes care of questions from patients on VKA. However, for NOACs, no safety network exists in this respect, bouncing all drug-related questions back to the prescriber. Therefore, the anticoagulation nurse will pick up all items regarding the NOAC resulting in a solid chain care to fill up this gap in our hospital.

## Limitations

From our observational data in this first cohort with short follow-up we cannot tell whether complex anticoagulation care pivoting around an anticoagulation nurse improves anticoagulation care and is cost-effective. However, considering the intensive early follow-up contacts of the anticoagulation nurse with the patients, leading to essential changes in therapy, this new example of ‘chain care’ seems to serve its goal. To support this notion, we can proudly state that thus far no major bleeding or stroke has occurred. At present we do not have data on quality of life but this may well improve through our service. Considering our experience up to now it seems advisable to install and expand anticoagulation care with the anticoagulation nurse as a pivot point.

## References

[1] Camm AJ, Kirchhof P, Lip GY (2010). Guidelines for the management of atrial fibrillation: the task force for the management of atrial fibrillation of the European Society of Cardiology (ESC). Europace.

[2] Camm AJ, Lip GY, De Caterina R (2012). Focused update of the ESC guidelines for the management of atrial fibrillation. An update of the 2010 ESC guidelines for the management of atrial fibrillation. Developed with the special contribution of the European Heart Rhythm Association. Europace.

[3] Werkgroep NOAC’s van de wetenschappelijke verenigingen en Orde van Medisch Specialisten. Leidraad begeleide introductie nieuwe orale antistollingsmiddelen.

[4] Landelijke stuurgroep keten antistollingsbehandeling. Landelijke standaard ketenzorg Antistolling voor de eerste- en tweedelijnszorg. www.knmp.nl. 2012.

[5] Heidbuchel H, Verhamme P, Alings M (2013). European Heart Rhythm Association practical guide on the use of new oral anticoagulants in patients with non-valvular atrial fibrillation. Europace.

[6] Harper P, Young L, Merriman E (2012). Bleeding risk with dabigatran in the frail elderly. N Engl J Med.

[7] Conolly SJ, Ezekowitz MA, Yusuf F (2009). Dabigatran versus warfarin in patients with atrial fibrillation. N Engl J Med.

[8] Patel MR, Mahaffey KW, Garg J (2011). Rivaroxaban versus warfarin in nonvalvular atrial fibrillation. N Engl J Med.

[9] Connolly SJ, Eikelboom J, Joyner C (2011). Apixaban in patients with atrial fibrillation. N Engl J Med.

[10] Dinh T, Baur LHB, Pisters R (2011). Feasibility of TEE-guided stroke risk assessment in atrial fibrillation-background, aims, design and baseline data of the TIARA pilot study. Neth Heart J.

